# Comprehensive Performance Regulation and Characterization of Polypropylene/Elastomer Composite Insulation Materials

**DOI:** 10.3390/polym17040530

**Published:** 2025-02-18

**Authors:** Xinhua Dong, Xianhao Fan, Wei Wang

**Affiliations:** 1 State Key Laboratory of Alternate Electrical Power System with Renewable Energy Sources, North China Electric Power University, Beijing 102206, China; wwei@ncepu.edu.cn; 2 School of Electrical Engineering, Tsinghua University, Beijing 100084, China; xianhao_fan@163.com

**Keywords:** elastomer, polypropylene, high-voltage cable, mechanical properties, electrical properties

## Abstract

Polypropylene (PP) represents an important development direction for recyclable cable insulation materials. This paper investigates the synergistic improvement mechanism of the mechanical and electrical properties of PP. The effect of elastomers on the aggregation structure and carrier migration of the samples was investigated by analyzing the microstructure, crystallization, and mechanical and electrical properties of PP/elastomer composites. The results show that elastomers can reduce the tensile modulus of PP, while improving the tensile strength and elongation at break, thereby enhancing the mechanical properties of PP. The elastomer and the PP matrix have better compatibility and introduce amorphous regions, generating a large number of crystalline–amorphous interfaces, which introduce deep traps and reduce the mobility of carriers. Meanwhile, with an increase in elastomer content, the breakdown strength and volume resistivity both increase and then decrease. Among the samples tested, PP/PBE-2 has the best electrical properties and is the insulation material with the best mechanical and electrical properties. This study provides an important reference for the comprehensive performance regulation of PP insulation materials for high-voltage cables.

## 1. Introduction

Cross-linked polyethylene (XLPE) has excellent mechanical and electrical properties and is the most widely used high-voltage (HV) cable insulation material [1,2]. However, XLPE is a thermosetting material, which makes it difficult to recycle after the cable is out of operation. Typically, it can only be incinerated, causing a serious waste of resources and environmental pollution, which is not in line with the development concept of green and environmental protection [3]. Therefore, developing new recyclable cable insulation materials has important practical significance. Polypropylene (PP) offers the advantages of simple processing technology, recyclability, and excellent insulation properties. These factors make it an important development direction for recyclable cable insulation materials in the future [4,5,6,7].

PP materials have a high tensile modulus, high low-temperature brittleness, and poor mechanical properties, which greatly limit the application scenarios of PP and make it difficult to use directly as an insulation material for cables [8]. For HV cable insulation materials, scholars have conducted extensive modification research. Commonly used modification methods for PP insulation materials include physical and chemical modifications [8,9,10]. Physical modification improves the properties of polymers by adding additives, mainly through nano-doping and blending modification. Chemical modification improves polymer properties by changing the molecular chain structure of the polymer and mainly includes copolymerization and grafting modifications. Compared with chemical modification, physical modification is simple and easy to implement, which can reduce the cost of PP modification.

Blending modification is a commonly used method in the physical modification of PP. It mainly involves melting PP and elastomer materials, which can make up for the weaknesses of single-component polymers and enhance the properties of polymer materials [4,11]. Currently, commonly used modified elastomers include ethylene propylene diene monomer (EPDM), polyolefin elastomer (POE), ethylene vinyl acetate copolymer (EVA), and hydrogenated styrene–butadiene block copolymer (SEBS). Studies have shown that blending modification with 40% EPDM elastomer can reduce the tensile strength of PP to 20 MPa but results in a 10% reduction in the DC breakdown strength, as well as a surge in the injection of space charge inside the sample [12]. The introduction of POE can significantly enhance the flexibility of PP and improve the mechanical properties of the material, but the volume resistivity and breakdown strength are reduced to a certain extent, and the accumulation of space charge also increases [13]. The improvement in the mechanical properties of PP by the EVA elastomer is not obvious enough, and at the same time, space charges easily accumulate under the direct current field [14]. The SEBS elastomer can improve the toughness and impact performance of PP, but leads to an increase in the accumulation of space charge inside the sample [15].

Studies have shown that blended elastomers can improve the flexibility and impact resistance of PP, significantly enhancing the mechanical properties of the material. However, this improvement leads to a reduction in electrical properties, such as breakdown strength, volume resistivity, and space charge. Currently, the elastomers used are unable to achieve synergistic improvement in the mechanical and electrical properties of PP cables, especially for HV PP cable insulation materials. Due to the better compatibility between propylene-based elastomer (PBE) and PP, PBE has more research value and potential in blending modification [16].

This paper selected PBE and PP for blending to prepare three groups of composites with different ratios. Firstly, the microstructure and crystallization characteristics of each sample were characterized. Then, the influence of blending modification on the mechanical and electrical properties of the samples was discussed. Finally, the influence mechanism of blending modification on the mechanical and electrical properties of the samples was analyzed. At present, the modification methods used for PP cannot achieve a synergistic improvement in mechanical and electrical properties. This is especially true for polypropylene cables, where achieving synergistic improvement in the mechanical and electrical properties of insulation materials is a key issue that needs to be urgently addressed. The research results provide a reference for the development of PP insulation materials with synergistic improvement in mechanical and electrical properties and the application of HV-grade PP cables.

## 2. Sample Preparation and Testing Methods

### 2.1. Material Preparation

Certain PP (melt flow rate: 3.7 g/10 min; density: 0.91 g/cm^3^; China Petroleum and Chemical Corporation, Beijing, China) and PBE (melt flow rate: 0.69 g/10 min; density: 0.87 g/cm^3^; propylene content: 90 wt%; China Petroleum and Chemical Corporation) particles were placed in a torque rheometer and preheated at 175 °C for 1 min and then blended at the same temperature for 20 min. At the end of blending, the rotation was stopped, and the PP/PBE blends were obtained. The PBE blending contents were 30%, 50%, and 70%, respectively, and the corresponding PP/PBE blends were named PP/PBE-1, PP/PBE-2, and PP/PBE-3, respectively.

Pellets of PP, PP/PBE-1, PP/PBE-2, and PP/PBE-3 were prepared as film samples. The prepared pellets were first heated in a mold at 200 °C for 5 min and then hot-pressed at 15 MPa for 10 min. The mold was subsequently cooled to room temperature under 10 MPa using a liquid-cooling system. Film samples with thicknesses of 100 μm and 200 μm were produced for subsequent testing.

### 2.2. Testing Methods

The blending effect of PBE was characterized by Fourier transform infrared spectroscopy (FTIR). The transmission mode was used in the test, with a resolution of 4 cm^−1^ and 16 scanning times. A scanning electron microscope (SEM) was used to characterize the microstructure after blending PBE. The spherulite morphology of each sample was observed using a polarizing microscope (POM). The crystal structure of the samples was characterized by X-ray diffraction (XRD). The thermal properties of each sample were investigated using differential scanning calorimetry (DSC).

The mechanical properties of each sample were tested using a tensile testing machine. The sample size was determined according to ISO 527-3:2018 [17], and tensile testing was carried out at a speed of 50 mm/min. A breakdown tester was used to test the DC breakdown strength of the samples. The conduction current of the samples was measured using a standard three-electrode system. Thermally stimulated depolarizing current (TSDC) spectra were measured to analyze the trap characteristics of the samples. 

## 3. Results

### 3.1. The Microstructure of PP/PBE Blends

**Figure 1:**
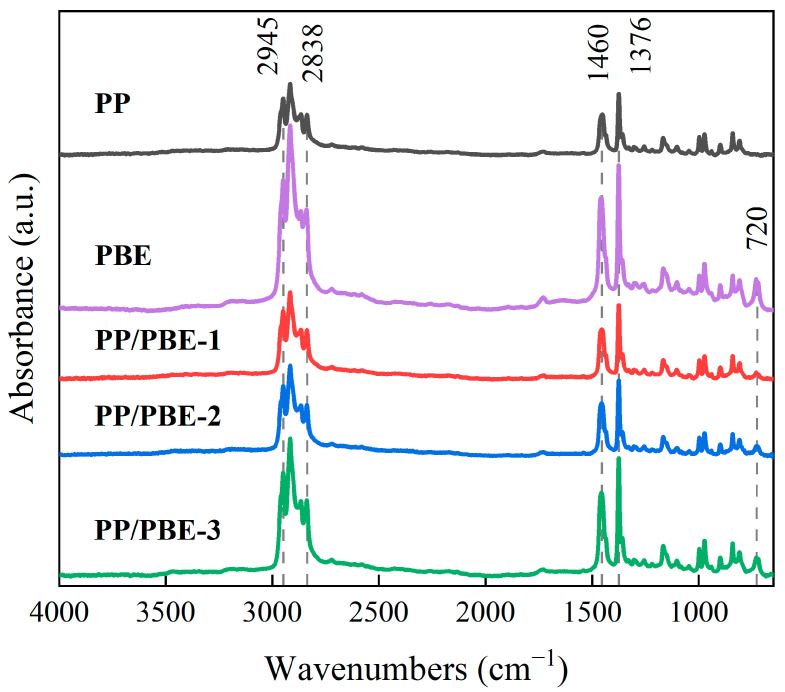
FTIR characterization of the samples.

Figure 1 shows the FTIR spectra. The absorption peaks of the curves between 2838 cm^−1^ and 2945 cm^−1^ correspond to the stretching vibration of PP methyl (CH_3_) and methylene (CH_2_) [18]. In addition, the absorption peaks at 1376 cm^−1^ and 1460 cm^−1^ of each curve correspond to the bending vibrations of PP methyl (CH_3_) [19].

The PP/PBE samples have similar FTIR spectra to PP, but a new absorption peak appears near 720 cm^−1^. The higher the PBE content, the higher the peak intensity. The absorption peak near 720 cm^−1^ corresponds to the vibration of the symmetrical C-H group of ethylene, indicating the presence of PBE [20,21].

**Figure 2:**
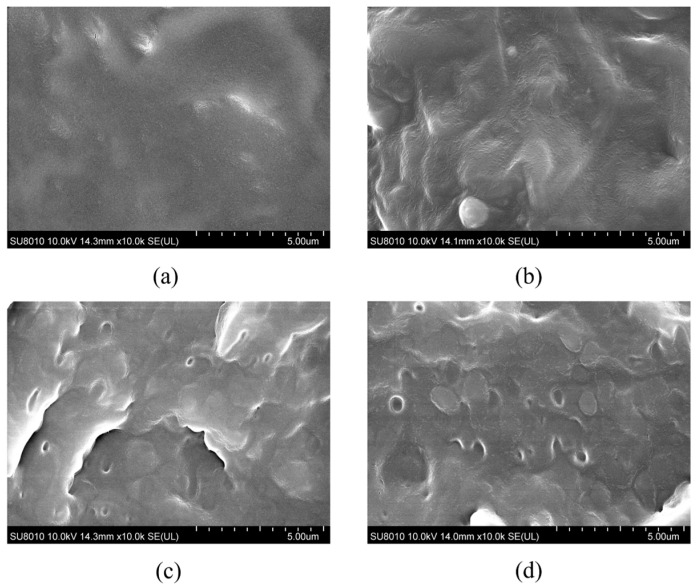
SEM images of (**a**) PP, (**b**) PP/PBE-1, (**c**) PP/PBE-2, and (**d**) PP/PBE-3.

In PP/elastomer composites, the dispersion state of the elastomer is closely related to its toughening effect. Figure 2 shows SEM images of each sample. For PP, due to its inherent brittleness, brittle fracture occurs when bending in liquid nitrogen. Its fracture surface is smooth and flat, as shown in Figure 2a. For PP/PBE samples, there is an obvious two-phase structure, in which one part is the PP matrix and the other part is PBE. The two-phase structure of the PP/PBE samples is a “sea–island structure”, in which the PP matrix is the “sea phase” and the PBE is the “island phase”. It can be found that in the PP/PBE samples, the PBE particles are uniformly dispersed in the PP matrix, and the elastomers are well dispersed with almost no agglomeration. This shows that there is better compatibility between PBE and PP [16].

**Figure 3:**
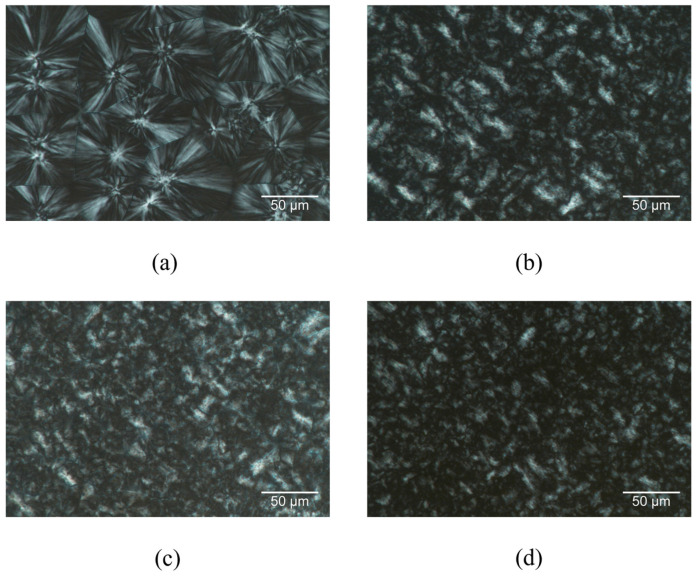
POM images of (**a**) PP, (**b**) PP/PBE-1, (**c**) PP/PBE-2, and (**d**) PP/PBE-3.

Figure 3 shows the spherulite morphology after the isothermal crystallization of each sample. The spherulites of PP are relatively perfect, with obvious boundaries and high isotacticity. For PP/PBE samples, the addition of PBE plays a role in heterogeneous nucleation, resulting in a sharp increase in the number of spherulites and a significant reduction in the size of spherulites compared with PP [22]. With the increase in PBE content, the number of spherulites in PP/PBE samples further increases, and the size of spherulites further decreases. The boundary between spherulites and the amorphous region further increases. This leads to an increase in the irregularity of the spherulite structure and a decrease in crystallinity [23].

**Figure 4:**
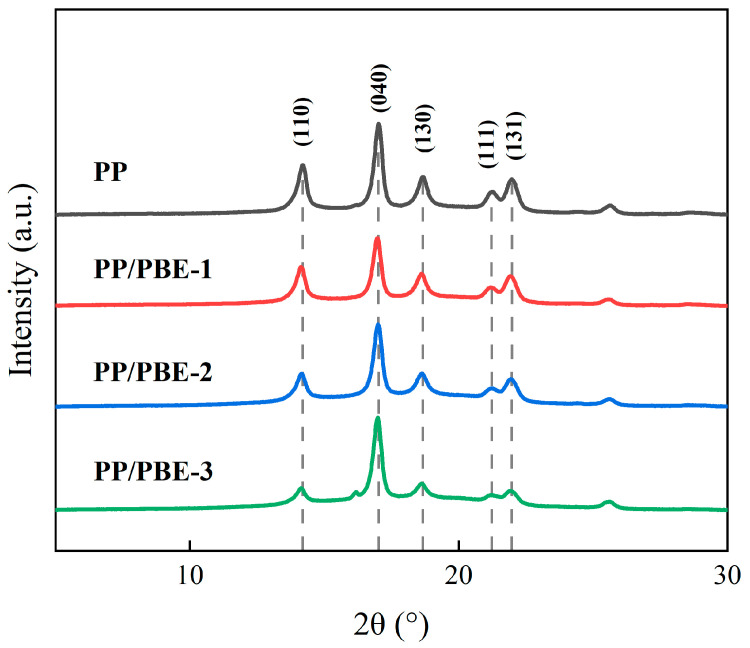
XRD patterns of the samples.

Figure 4 shows the XRD patterns of each sample. Each sample exhibits diffraction peaks at 2θ = 14.18°, 16.99°, 18.64°, 21.26°, and 21.96°, respectively, corresponding to the (110), (040), (130), (111), and (131) crystal planes of the α-type of PP [24]. The diffraction peaks of PP/PBE samples are the same as those of PP, indicating that the blending modification does not significantly change the crystal form of PP material. The results indicate that compared with PP, PBE blending affects the spherulite morphology and crystallinity of PP but does not affect the crystal structure of PP [25].

According to the XRD curves in Figure 4, the crystal structure parameters of PPH and the PP/PBE samples were calculated, as shown in Table 1. The interplanar spacing (*d*) is negatively correlated with the incident angle θ, and the smaller the θ, the larger the interplanar spacing. According to Table 1, it can be observed that the diffraction angle of the diffraction peak of the PP/elastomer composites shows a gradually decreasing trend with the increase in PBE, and the interplanar spacing shows a gradually increasing trend. This indicates that the interplanar spacing of PP/elastomer composites increases with the introduction of PBE. The full width at half maxima (FWHM) of the diffraction peaks can reflect the degree of perfection of polymer crystallization. With the increase in PBE, the FWHM also shows an increasing trend, indicating that the degree of perfection of PP/elastomer composites deteriorates.

### 3.2. Thermal Properties of PP/PBE Blends

**Figure 5:**
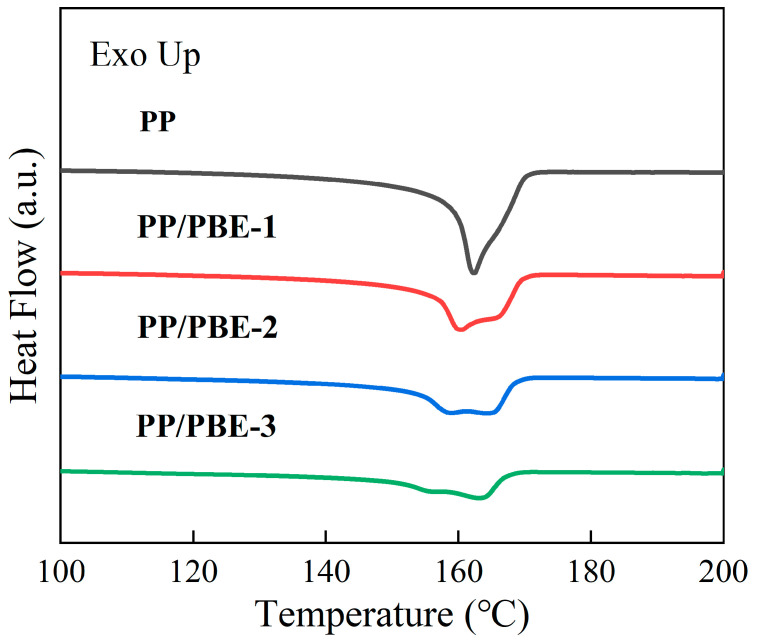
Melting curve spectra of each sample.

**Figure 6:**
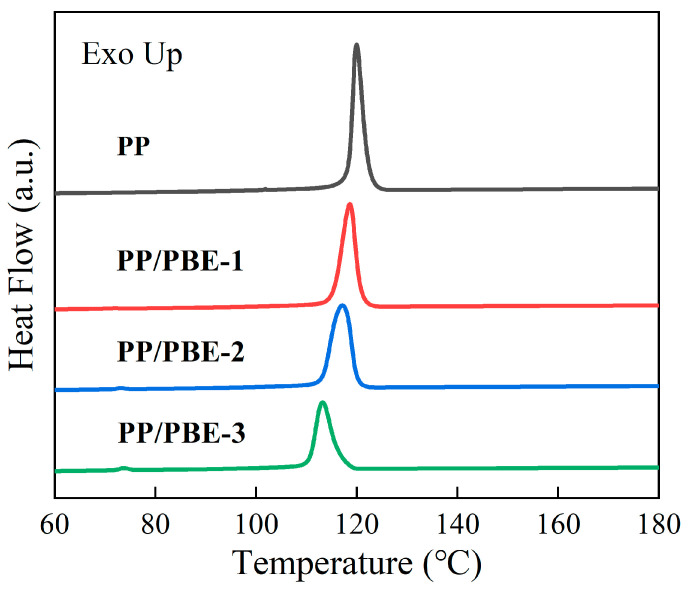
Crystallization curve spectra of each sample.

The thermal properties of each sample are shown in Figure 5 and Figure 6, and the thermal property parameters of each sample are shown in Table 2. The melting temperature (*T*_m_) and crystallization temperature (*T*_c_) represent the peak temperature of the polymer during endothermic and exothermic processes, respectively. The crystallinity (*X*_c_) was calculated based on the melting enthalpy of the polymer and the melting enthalpy of the completely crystalline polymer (209 J/g). The results show that compared with PP, the *T*_m_ of PP/PBE samples decreased to a certain extent, and the higher the PBE content, the greater the decrease in *T*_m_. Among the samples, the largest decrease in PP/PBE-3 is 3.85%, and the smallest decrease in PP/PBE-1 is only 1.15%. This change in *T*_m_ is due to the presence of the elastomer, which disrupts the continuity of the propylene unit and inhibits crystallization, thus reducing the *T*_m_ of the sample [26].

The *T*_c_ of each sample also shows the same trend as *T*_m_. The *X*_c_ of PP/PBE samples was lower than that of PP, and the higher the PBE content, the smaller the *T*_c_. This is because the increase in PBE content inhibits the crystallization process of PP and reduces the *T*_c_ of PP [27].

Compared with PP, the *X*_c_ of PP/PBE samples also decreased with the increase in PBE content. On the one hand, with the increase in PBE content, the thickness of the PP crystals in PP/PBE samples decreases significantly. On the other hand, the size of the elastomer increases with the increase in PBE content, which disrupts the orderly arrangement of surrounding molecular chains, leading to a further decrease in *X*_c_ [27,28].

### 3.3. Mechanical Properties

**Figure 7:**
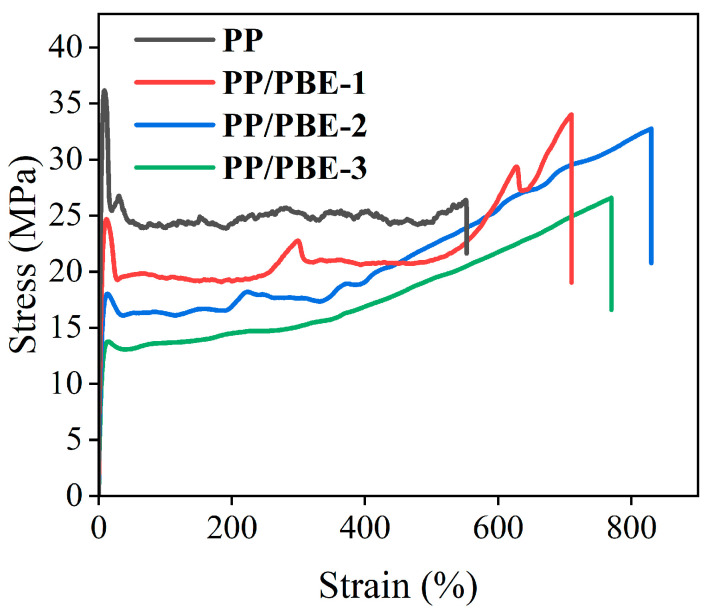
Stress–strain curves of each sample.

**Figure 8:**
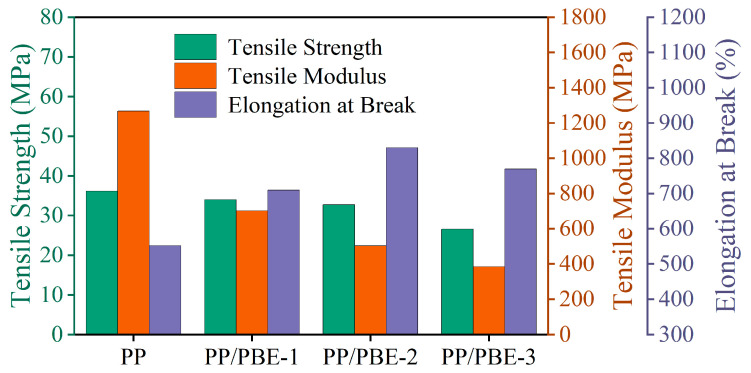
Mechanical property parameters of each sample.

Tensile testing was performed on each sample to evaluate mechanical properties. Figure 7 shows the stress–strain curves of each sample. Each sample presents a typical elastic–plastic stress–strain curve, with the corresponding mechanical property parameters shown in Figure 8. Compared with PP, the tensile modulus of PP/PBE samples gradually decreased with the increase in PBE content. Blending PBE can significantly improve the high tensile modulus of PP, where the lowest tensile modulus of PP/PBE-3 is 385.14 MPa, which is 69.64% lower than that of PP. The elongation at break of PP can be significantly improved by blending PBE. PP/PBE-2 has the highest elongation at break of 830.02%, which is 50.26% higher than that of PP. However, with the increase in PBE content, the elongation at break decreases, indicating that excessive PBE blending is not conducive to the enhancement of the mechanical properties of PP. With the increase in PBE content, the tensile strength decreases but still remains at a high level. This shows that blending PBE can enhance the modulus and elongation at break of PP while maintaining high tensile strength, which is conducive to improvement in the mechanical properties of PP.

### 3.4. DC Breakdown Strength

**Figure 9:**
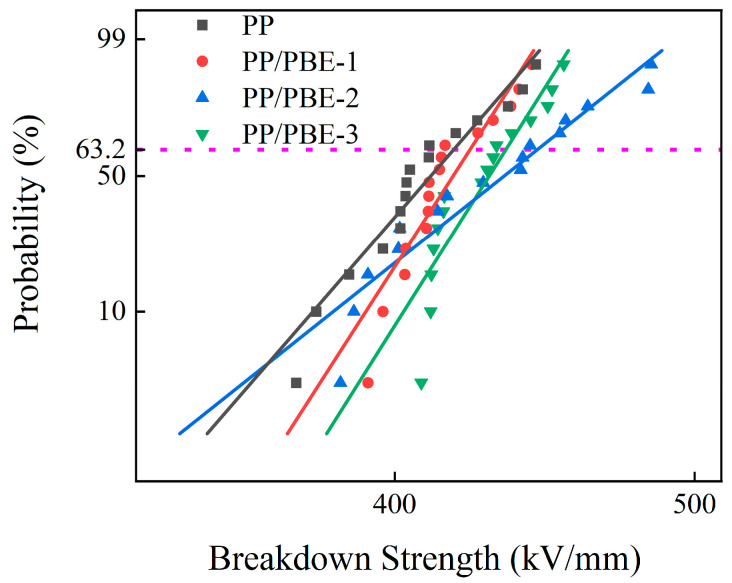
Breakdown strength of the samples.

The Weibull distribution of DC breakdown strength for each sample is shown in Figure 9. The test temperature was room temperature, and the ramp rate was 1 kV/s. The breakdown strength was analyzed using the following equation [29]:

(1) $$F(E)=1-\exp(-{\left(E/\alpha \right)}^{\beta })$$

where *E* is the tested breakdown strength, *α* is a scale parameter representing the characteristic breakdown strength at a failure probability of 63.2%, and *β* is a shape parameter representing the dispersion of the measured values.

The *α* and *β* values of each sample are presented in Table 3. The characteristic breakdown strengths (*α*) of PP and PP/PBE samples are 417.85 kV/mm, 423.01 kV/mm, 444.71 kV/mm, and 434.45 kV/mm, respectively. The *β* of each sample is greater than 10, indicating that the preparation of the sample is uniform and stable. With the increase in PBE content, the breakdown strength of PP/PBE samples increases and then decreases. It can be found that PP/PBE-2 exhibits the highest breakdown strength, which is 6.43% higher than that of PP, while that of PP/PBE-3 is 2.31% lower than that of PP/PBE-2. Therefore, too high a PBE content can reduce the breakdown strength of PP, which is detrimental to improvement in the electrical properties of PP.

### 3.5. Conductivity

**Figure 10:**
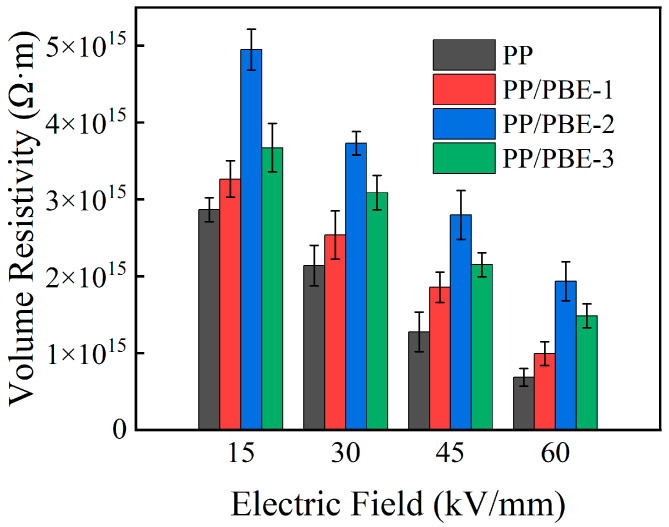
Volume resistivity of the samples.

Figure 10 shows the volume resistivity test results of each sample. Compared with the PP/PBE samples, the volume resistivity of PP is the lowest under each electric field. Meanwhile, the volume resistivity of each sample decreases with the increase in the electric field, and the highest volume resistivity of PP is 3.86 × 10^15^ Ω·m. With the increase in PBE content, the volume resistivity of PP/PBE samples increases and then decreases. The highest volume resistivity of PP/PBE-2 is 4.95 × 10^15^ Ω·m, which is 1.72 times that of PP. The trend in volume resistivity is consistent with that in breakdown strength, indicating that too high a content of PBE can reduce the electrical properties of PP, which is detrimental to improvement in the comprehensive performance of PP.

## 4. Discussion

The stress–strain curve of polymers is mainly divided into a linear change stage, cold drawing stage, and strain hardening stage. The tensile modulus of the polymer depends on the linear change stage. Due to the better flexibility of the ethylene molecular chain, the tensile modulus of the PP/PBE samples decreases significantly. The higher the PBE content, the more the tensile modulus decreases. The tensile strength and elongation at break of the polymer depend on the cold drawing stage and the strain hardening stage. The strain during cold drawing mainly comes from the slip between polymer lamellar crystals. Increasing the content of PBE can promote the slip between lamellar crystals, reduce tensile strength, and increase elongation at break. However, adding excessive PBE can lead to a decrease in the crystallinity of the polymer and an oversized elastomer. This reduces the degree of slip in the crystalline regions during the stretching process. As a result, different deformation quantities occur between the PP matrix and the elastomer. Therefore, it is easy to generate micropores at the interface, leading to premature fracture, which in turn leads to a decrease in tensile strength and elongation at break [30].

**Figure 11:**
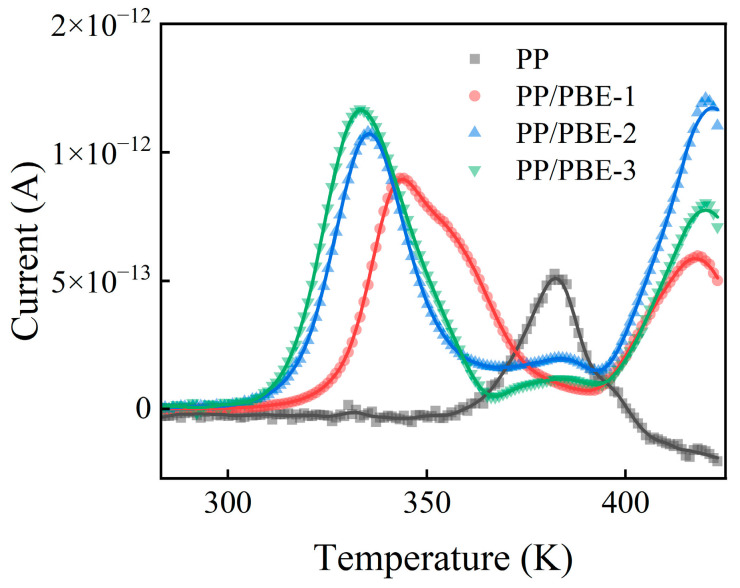
TSDC curves of the samples.

**Figure 12:**
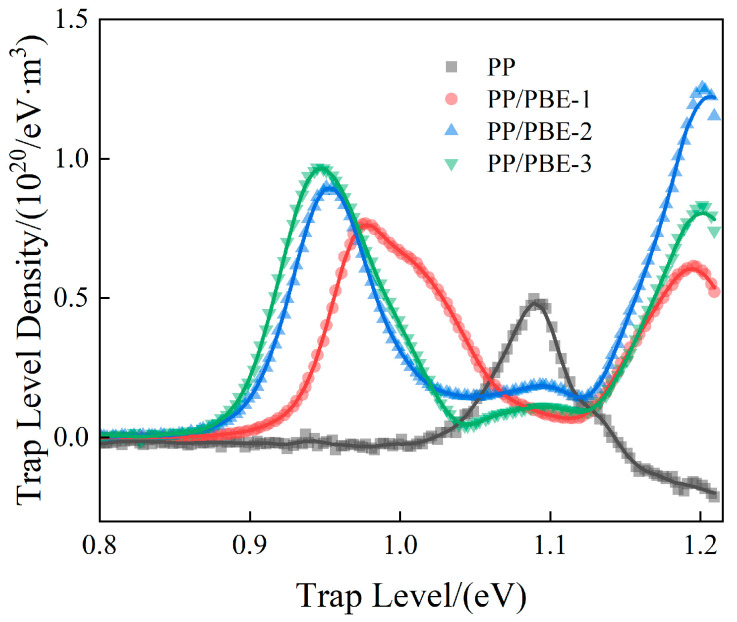
Trap distribution of the samples.

The electrical properties of PP/PBE samples are different from those of PP and are closely related to carrier injection, migration, and accumulation. In order to analyze the trap characteristics of each sample, the TSDC curves of each sample were tested, as shown in Figure 11. According to the TSDC measurement results, the trap level distribution of each sample was calculated [31], as shown in Figure 12.

It can be found that there are two trap level centers located at about 0.96 eV and 1.2 eV in PP/PBE samples, corresponding to shallow and deep traps, respectively. With the increase in PBE content, both the deep trap level and density of PP/PBE samples increase and then decrease. Compared with PP, PP/PBE-2 has the highest trap level and trap density, which increase from 1.09 eV and 0.50 × 10^20^ eV^−1^m^−3^ to 1.21 eV and 1.25 × 10^20^ eV^−1^m^−3^, respectively. Therefore, an appropriate content of PBE can significantly increase the trap level and density of PP/PBE samples.

PP is a semi-crystalline polymer. It is generally believed that deep traps mainly come from the boundaries of crystalline and amorphous regions, while shallow traps come from amorphous regions [32]. Therefore, chemical defects introduce deep traps, while physical defects introduce shallow traps. For PP/PBE samples, the density of shallow traps increases with the increase in PBE content, which is closely related to the change in crystallinity. The decrease in crystallinity leads to an increase in amorphous region content, and structural defects lead to an increase in physical defects, which in turn leads to an increase in the density of shallow traps [33]. For deep traps, compared with PP, PP/PBE-2 has the highest deep trap level and density, which is not consistent with the trend of crystallinity. With the increase in PBE content, the spherulite size of the polymer decreases, and the number of elastomer microspheres increases. Therefore, the increase in PBE content leads to more boundaries between the crystalline and amorphous regions in the polymer. The number of spherulite defects increases, and deeper traps are introduced around the spherulites. The density of deep traps also greatly increases, which hinders charge transport [34]. Compared with PP/PBE-2, although PP/PBE-3 has more interfaces, its crystallinity is greatly reduced, and the elastomer volume inside the sample is larger. As a result, its trap level and density are greatly reduced, providing a diffusion channel that carriers can easily penetrate, leading to the significant deterioration of electrical properties [35]. In summary, PP/PBE-2 has the most excellent electrical properties while greatly improving the mechanical properties of PP. It is an insulation material that achieves synergistic improvement in mechanical and electrical properties.

## 5. Conclusions

In this paper, the influence and mechanism of elastomer on the mechanical and electrical properties of PP insulation material for HV cables were investigated, and the optimal PP/PBE sample for HV cable insulation material was determined. The conclusions are as follows:

(1) The elastomer and the PP matrix inside the PP/PBE sample show a “sea–island structure”. The blending modification does not change the crystal form of PP, and the α-type remains dominant in PP/PBE samples. The crystallinity of the samples gradually decreases with the increase in ethylene content;
(2) PBE and the PP matrix have better compatibility and significantly improve the mechanical properties of PP. Compared with PP, the tensile modulus of PP/PBE-2 decreases by 60.29%, and the elongation at break increases by 50.26% while still maintaining high tensile strength;
(3) With the increase in PBE content, the breakdown strength and volume resistivity of PP/PBE samples increase and then decrease. The characteristic breakdown strength and volume resistivity of PP/PBE-2 are the highest, at 444.71 kV/mm and 4.95 × 10^15^ Ω·m, respectively, which represent increases of 6.43% and 72.47% compared with PP. The results show that an appropriate content of PBE can significantly enhance the insulation properties of PP/PBE samples;
(4) A large number of boundaries between the crystalline and amorphous regions is generated in PP by blending elastomer, which can enter many deep traps. An appropriate content of PBE can balance the relationship between crystallinity and trap level and density, reduce carrier migration, hinder charge transport, and improve the electrical properties of the material.

This research shows that PP/PBE-2 can achieve synergistic improvement in the mechanical and electrical properties of PP cable insulation materials. This provides a theoretical basis and reference for the development and application of HV cable insulation materials.

## Figures and Tables

**Table 1:** Crystal structure parameters of the samples.

Samples	Crystal Planes	2θ (°)	*d* (Å)	FWHM
PP	(110)	14.20	6.23	0.405
	(040)	17.02	5.21	0.348
	(130)	18.68	4.75	0.394
	(111)	21.24	4.18	0.220
	(131)	21.98	4.04	0.517
PP/PBE-1	(110)	14.16	6.25	0.408
	(040)	17.00	5.21	0.349
	(130)	18.64	4.76	0.420
	(111)	21.24	4.18	0.295
	(131)	21.94	4.05	0.532
PP/PBE-2	(110)	14.14	6.26	0.423
	(040)	17.00	5.21	0.353
	(130)	18.62	4.76	0.420
	(111)	21.20	4.19	0.445
	(131)	21.92	4.05	0.545
PP/PBE-3	(110)	14.12	6.27	0.434
	(040)	17.98	5.22	0.363
	(130)	18.62	4.76	0.448
	(111)	21.20	4.19	0.456
	(131)	21.88	4.06	0.593

**Table 2:** Thermal performance parameters of each sample.

Samples	*T*_m_ (°C)	*T*_c_ (°C)	*X*_c_ (%)
PP	162.35	120.69	40.04
PP/PBE-1	160.48	118.45	32.67
PP/PBE-2	159.11	117.04	27.07
PP/PBE-3	156.18	113.68	19.25

**Table 3:** Breakdown strength parameters for the samples.

Samples	*α* (kV/mm)	*β*
PP	417.85	21.51
PP/PBE-1	423.01	29.03
PP/PBE-2	444.71	14.82
PP/PBE-3	434.45	29.56

## Data Availability

Data are contained within the article.

## References

[1] Du Y., Zhang H., Han W., Du X., Shang Y., Yang H., Wang X., Chen Q., Li Z. (2024). The network construction of a new byproduct-free XLPE-based insulation using a click chemistry-type reaction and a theoretical study of the reaction mechanism. Polymers.

[2] Wu Z.-Y., Jin Y.-Z., Shi Z.-X., Wang Z.-Y., Wang W. (2024). Study on the relationship between electron transfer and electrical properties of XLPE/modification SR under polarity reversal. Polymers.

[3] He J., Chen G. (2017). Insulation materials for HVDC polymeric cables. IEEE Trans. Dielectr. Electr. Insul..

[4] Pourrahimi A.M., Mauri M., D’Auria S., Pinalli R., Müller C. (2024). Alternative concepts for extruded power cable insulation: From thermosets to thermoplastics. Adv. Mater..

[5] Zhou Y., Dang B., Wang H., Liu J., Li Q., Hu J., He J. (2018). Polypropylene-based ternary nanocomposites for recyclable high-voltage direct-current cable insulation. Compos. Sci. Technol..

[6] Zhan Y., Yang X., Yang J., Hou S., Fu M. (2023). Improved electrical properties of organic modified thermoplastic insulation material for direct current cable application. Polymers.

[7] Zhang C., Shi W., Wang Q., Diao M., Hiziroglu H.R. (2021). Electrical property of polypropylene films subjected to different temperatures and DC electric fields. Polymers.

[8] Huang X., Zhang J., Jiang P., Tanaka T. (2020). Material progress toward recyclable insulation of power cables part 2: Polypropylene-based thermoplastic materials. IEEE Electr. Insul. Mag..

[9] Zhou Y., Peng S., Hu J., He J. (2017). Polymeric insulation materials for HVDC cables: Development, challenges and future perspective. IEEE Trans. Dielectr. Electr. Insul..

[10] Li Z., Du B. (2018). Polymeric insulation for high-voltage DC extruded cables: Challenges and development directions. IEEE Electr. Insul. Mag..

[11] AlEidan K.E., Kenawy E.-R., Mandour H.S.A., Azaam M.M., AlOtaibi B.S. (2024). Improving of impact copolymer PP (ICP) blends properties via compounding. Polym. Bull..

[12] Hosier I.L., Vaughan A.S., Swingler S.G. (2011). An investigation of the potential of polypropylene and its blends for use in recyclable high voltage cable insulation systems. J. Mater. Sci..

[13] Zhou Y., He J., Hu J., Huang X., Jiang P. (2015). Evaluation of polypropylene/polyolefin elastomer blends for potential recyclable HVDC cable insulation applications. IEEE Trans. Dielectr. Electr. Insul..

[14] Ramírez-Vargas E., Navarro-Rodríguez D., Huerta-Martínez B.M., Medellín-rodríguez F.J., Lin J.S. (2000). Morphological and mechanical properties of polypropylene [PP]/poly(ethylene vinyl acetate) [EVA] blends. I. homopolymer PP/EVA systems. Polym. Eng. Sci..

[15] Zha J.-W., Wang J.-F., Wang S.-J., Qin Q., Dang Z.-M. (2018). Effect of modified ZnO on electrical properties of PP/SEBS nanocomposites for HVDC cables. IEEE Trans. Dielectr. Electr. Insul..

[16] Gao Y., Li J., Chen G., Han T., Du B. (2020). Compatibility dependent space charge accumulation behavior of polypropylene/elastomer blend for HVDC cable insulation. IEEE Trans. Dielectr. Electr. Insul..

[17] (2018). Plastics—Determination of Tensile Properties. Part 3: Test Conditions for Films and Sheets.

[18] Azmi A., Lau K.Y., Ahmad N.A., Abdul-Malek Z., Tan C.W., Ching K.Y., Vaughan A.S. (2021). Structure-dielectric property relationship in polypropylene/multi-element oxide nanocomposites. IEEE Trans. Nanotechnol..

[19] Fang J., Zhang L., Sutton D., Wang X., Lin T. (2012). Needleless melt-electrospinning of polypropylene nanofibres. J. Nanomater..

[20] Rahim N.H., Lau K.Y., Muhamad N.A., Mohamad N., Rahman W.A.W.A., Vaughan A.S. (2019). Effects of filler calcination on structure and dielectric properties of polyethylene/silica nanocomposites. IEEE Trans. Dielectr. Electr. Insul..

[21] Azrin N.A., Ahmad N.A., Lau K.Y., Kamarudin S.N.H. (2024). Structure, mechanical, and dielectric properties of polypropylene blended with ethylene and propylene-based elastomers. J. Appl. Polym. Sci..

[22] Du B., Liu H., Li Z. (2024). Effect of nucleating agent and cooling rate on dielectric Property of PP/POE cable insulation. IEEE Trans. Dielectr. Electr. Insul..

[23] Chen R., Shangguan Y., Zhang C., Chen F., Harkin-Jones E., Zheng Q. (2011). Influence of molten-state annealing on the phase structure and crystallization behaviour of high impact polypropylene copolymer. Polymer.

[24] Lezak E., Bartczak Z., Galeski A. (2006). Plastic deformation of the γ phase in isotactic polypropylene in plane-strain compression. Macromolecules.

[25] Luo G., Liu G., Chen Y., Liang W., Liu G., Niu Y., Li G. (2018). High performance glass fiber reinforced polypropylene realized by reactive extrusion technology. Compos. Sci. Technol..

[26] Sangroniz L., Cavallo D., Santamaria A., Müller A.J., Alamo R.G. (2017). Thermorheologically complex self-seeded melts of propylene–ethylene copolymers. Macromolecules.

[27] Huang X., Fan Y., Zhang J., Jiang P. (2017). Polypropylene based thermoplastic polymers for potential recyclable HVDC cable insulation applications. IEEE Trans. Dielectr. Electr. Insul..

[28] Yang K., Ren Y., Wu K., Li J., Jing Z., Zhang Z., Dong J.-Y. (2022). Enhancing electrical properties of impact polypropylene copolymer for eco-friendly power cable insulation by manipulating the multiphase structure through molten-state annealing. Compos. Sci. Technol..

[29] Zhou Y., Hu J., Dang B., He J. (2016). Mechanism of highly improved electrical properties in polypropylene by chemical modification of grafting maleic anhydride. J. Phys. D Appl. Phys..

[30] Yang K., Liu Y., Yan Z., Tian Y., Liu Y., Jing Z., Li J., Li S. (2020). Enhanced morphology-dependent tensile property and breakdown strength of impact polypropylene copolymer for cable insulation. Materials.

[31] Tian F., Bu W., Shi L., Yang C., Wang Y., Lei Q. (2011). Theory of modified thermally stimulated current and direct determination of trap level distribution. J. Electrostat..

[32] Li Z., Zhong Z., Du B. (2019). Dielectric relaxation and trap-modulated DC breakdown of polypropylene blend insulation. Polymer.

[33] Peng S., Dang B., Zhou Y., Hu J., He J. (2016). Functionalized TiO_2_ nanoparticles tune the aggregation structure and trapping property of polyethylene nanocomposites. J. Phys. Chem. C.

[34] Dang B., He J., Hu J., Zhou Y. (2016). Large improvement in trap level and space charge distribution of polypropylene by enhancing the crystalline–amorphous interface effect in blends. Polym. Int..

[35] Li X., Du Q., Kang J., Tu D. (2002). Influence of microstructure on space charges of polypropylene. J. Polym. Sci. B Polym. Phys..

